# The Exploration of Poor Ovarian Response–Related Risk Factors: A Potential Role of Growth Differentiation Factor 8 in Predicting Ovarian Response in IVF-ET Patient

**DOI:** 10.3389/fendo.2021.708089

**Published:** 2021-09-24

**Authors:** Long Bai, Huihui Pan, Yinjun Zhao, Qingqing Chen, Yu Xiang, Xiaohang Yang, Yimin Zhu

**Affiliations:** ^1^ Department of Reproductive Endocrinology, Women’s Hospital, School of Medicine, Zhejiang University, Hangzhou, China; ^2^ Key Laboratory of Reproductive Genetics (Ministry of Education) and Women’s Reproductive Health Laboratory of Zhejiang Province, Women’s Hospital, Zhejiang University School of Medicine, Hangzhou, China; ^3^ Institute of Genetics and Department of Genetics, School of Medicine, Zhejiang University, Hangzhou, China

**Keywords:** receptor, ART, risk factor, poor ovarian response, GDF-8

## Abstract

Controlled ovarian hyperstimulation (COH) is the most common therapeutic protocol to obtain a considerable number of oocytes in IVF-ET cycles. To date, the risk factors affecting COH outcomes remain elusive. Growth differentiation factor 8 (GDF-8), a member of transforming growth factor β (TGF-β) superfamily, has been long discerned as a crucial growth factor in folliculogenesis, and the aberrant expression of GDF-8 is closely correlated with the reproductive diseases. However, less is known about the level of GDF-8 in IVF-ET patients with different ovarian response. In the present study, the potential risk factors correlated with ovarian response were explored using logistic regression analysis methods. Meanwhile, the expression changes of GDF-8 and its responsible cellular receptors in various ovarian response patients were determined. Our results showed that several factors were intensely related to poor ovarian response (POR), including aging, obesity, endometriosis, surgery history, and IVF treatment, while irregular menstrual cycles and PCOS contribute to hyperovarian response (HOR). Furthermore, POR patients exhibited a decrease in numbers of MII oocytes and available embryos, thereby manifesting a lower clinical pregnancy rate. The levels of GDF-8, ALK5, and ACVR2B in POR patients were higher compared with those in control groups, whereas the expression level of ACVR2A decreased in poor ovarian response patients. In addition, clinical correlation analysis results showed that the concentration of GDF-8 was negatively correlated with LH and estradiol concentration and antral follicle count. Collectively, our observations provide a novel insight of ovarian response–associated risk factors, highlighting the potential role of GDF-8 levels in ovarian response during COH process.

## Introduction

Infertility is a global issue, and its incidence of fertile population shows a rampantly increasing trend nowadays. Since the advent of *in vitro* fertilization embryo transfer (IVF-ET) in 1978, IVF-ET technology has been adopted worldwide as an approach to overcome infertility problem in couples seeking medical assistance. With IVF technology undergoing several developmental stages, controlled ovarian hyperstimulation (COH) has gradually become a main therapeutic approach to promote ovulation and subsequently obtain an optimum number of mature oocytes. Clinically, the effect of COH relies on the ovarian response, which is divided into poor, normal, and hyper response (1, 2). The poor ovarian response (POR) likely leads to few retrieved oocytes and diminished clinical pregnancy rate (3), whereas the pathogenesis of which has not yet been clearly clarified. Owing to the absence of reliable markers for direct prediction, it is difficult for clinicians to assess the ovarian response of women undergoing COH. Instead, ovarian reserve, referred to as reflection of fertility performance, is utilized as an indirect marker to clinically evaluate the ovarian response of patients during the process of assisted reproductive technology (ART) (4). Hyper ovarian response (HOR) is another pathologic condition in COH. In the last few years, it is suggested by clinical investigations that patients with HOR have an increasing tendency to develop ovarian hyperstimulation syndrome (OHSS) (5). Thus, the precise prediction of HOR would be beneficial to preventing the occurrence of OHSS in ART. To date, multiple factors have been reported to act in parallel regarding ovarian response, in terms of age, body mass index (BMI), genetics, environment, etc. Nevertheless, it still seems challenging to precisely assess ovarian response due to the lack of valid markers. Therefore, exploration of ovarian response–related risk factors would be advantageous to the evaluation of ovarian function and ideal IVF outcomes.

The maintenance of a well-balanced follicular environment is essential for the follicle development. Cell–cell interaction, especially the coordinate crosstalk between the oocyte and its surrounding granulosa cells, plays a vital role in creating intrafollicular microenvironment suitably equipped for further maturation. Past experiments have shed the light on a variety of growth factors as active participants in the regulation of gamete-somatic cell communications. Growth differentiation factor 8 (GDF-8), belonging to transforming growth factor β (TGF-β) superfamily, has been indicated to be profoundly involved in the modulation of folliculogenesis (6). Results of immunohistochemistry analysis illustrated that GDF-8 is widely expressed in different size of human growing follicles (7). In addition, further clinical studies reveal the dynamic changes of serum GDF-8 during the COH process, suggesting a changing role of GDF-8 in the progressively different stages of folliculogenesis (8). Within the growth phase of follicle development, GDF-8 mediates the response of granulosa cell to gonadotrophins followed by taking an active part in ovarian steroidogenesis (9). Meanwhile, *in vitro* experiment demonstrates that GDF-8 inhibits the proliferation of human granulosa cells (10). Considered as a substantial modulator in ECM remodeling and cumulus-oocytes complex (COC) expansion, GDF-8 has been revealed as a potential element in the pathogenesis of polycystic ovary syndrome (PCOS) *via* clinical research (7, 8, 11, 12). Notably, two of the latest studies found that the concentrations of GDF-8 in follicular fluid is negatively correlated with IVF and pregnancy outcomes, which illuminate the possibility of GDF-8 to assess the ovarian function (8, 11). Accordingly, we hypothesize that GDF-8 tends to be a potential predictor to evaluate the ovarian response in COH process. In the present study, we explored the ovarian response–related risk factors and determined the expression of GDF-8 and its responsible receptors expression in different ovarian response patients during COH.

## Materials and Methods

### Ethics Statement and Human Subjects

The study was approved by the Ethical Committee of the Women’s Hospital, Zhejiang University School of Medicine, China (File No. 20180141). All participants signed a document of informed consent before participation of the study. All the subjects were obtained from 767 women (166 POR, 409 normal response, and 192 HOR) undergoing IVF-ET treatment in the Reproductive Center of Women’s Hospital, School of Medicine, Zhejiang University from 2014 to 2015. The causes of infertility included fallopian tube factors, endometriosis, polycystic ovary syndrome, male factors, unexplained infertility, etc.

### Controlled Ovarian Hyperstimulation Protocol

Based on patient’s age, AFC, and basal endocrine condition, GnRHa long protocol or short protocol was selected. The long protocol was to start the intradermal injection of GnRHa in the middle of the luteal phase, which was the 21st day of menstrual cycle or the 7th day after the basal body temperature rose. On the 3rd day of the treatment cycle, daily intramuscular injection of rFSH (Gonal-F, Serono, Switzerland) was carried out, and Follicle monitoring under B ultrasound was started on the 8th day. When the average diameter of the largest follicles reached 18 mm or the average diameter of three follicles reached 16 mm, treatment of rFSH was halted and instead injection of 10,000 IU human chorionic gonadotropin (HCG, Organon, USA) was implemented. After 34–36 h, oocytes were collected by vaginal ultrasound-guided puncture. In short protocol, GnRHa was given from the second day of menstrual cycle, and rFSH was added from the third day of the treatment cycle. Recombinant follicle-stimulating hormone (FSH) was applied as gonadotropin, and the initial dose was determined according to the patient’s age, the number of antral follicles, and the previous response to gonadotropin. The following dose was adjusted based on follicle development and E2 monitoring. When the average diameter of the largest follicle reached 18 mm or the average diameter of three follicles reached 16 mm, the following treatment was performed as in long protocol described above.

### Diagnosis of Ovarian Response

#### Poor Ovarian Response

(1) Advanced maternal age (≥40 years old) or any other POR risk factor; (2) A previous POR with oocytes obtained by conventional stimulation ≤3; (3) Abnormal ovarian reserve test (ORT) result in terms of antimüllerian hormone (AMH) <0.5–1.1 ug/L or antral follicle count (AFC) <5–7. Two of these three criteria are required for a POR diagnosis. In addition, two episodes of POR after maximal stimulation are sufficient to define a patient as poor responder in the absence of advanced maternal age or abnormal POR.

#### Hyper Ovarian Response

Serum estradiol (E2) >11,010 pmol/L (3,000 pg/m1) on human chorionic gonadotrophin (hCG) given day or number of eggs obtained >15 or 20 when the Gn dosage is ≤225 U/d.

### IVF-ET

The obtained oocytes were fertilized *in vitro* according to conventional methods. After 18 h, the pronucleus and polar bodies were observed to evaluate the fertilization. Observation of embryo division was done on the second and third days after egg retrieval. Blastomere with regular morphology and less than 15% of fragment was regarded as a valid embryo. In the 4–5 weeks after embryo transfer, clinical pregnancy was diagnosed if the gestational sac was detected by vaginal ultrasound, or the ectopic pregnancy was confirmed by surgical pathology. Patients were diagnosed as biochemical pregnancy when HCG elevated but no embryo sac was observed. The canceled cycle was defined as the absence of transplantable embryos or the cancelation of the fresh embryo transfer due to OHSS.

### Enzyme-Linked Immunosorbent Assay Measurement

The GDF-8 Quantikine ELISA Kits (DGDF80) were obtained from R&D System (MN, USA). The serum or follicular fluid samples were collected at the day of oocyte retrieval and storage at −80°C if not detected immediately. The concentrations of GDF-8 in serum or follicular fluid were determined by enzyme-linked immunosorbent assay (ELISA) analysis according to the manufacturer’s instructions. The sensitivity of GDF-8 Quantikine ELISA Kit was 5.32 pg/ml. The intra- and inter-assay errors of the GDF-8 ELISA were 5.4 and 6, respectively.

### Western Blot

After oocytes were retrieved, the human granulosa-lutein (hGL) cells were purified from follicular fluid mixture by using density centrifugation from follicular aspirates. The cells were lysed, and protein concentration of sample was examined. Total 20 ug protein were loaded and separated using sodium dodecyl sulfate polyacrylamide gel electrophoresis (SDS-PAGE). After that, the proteins were transferred onto polyvinylidene difluoride (PVDF) membranes (Bio-Rad, USA) and blocked by Tris-buffered saline (TBS) containing 5% non-fat dry milk for 1 h at room temperature and incubated overnight at 4°C with GDF-8 (ab203076, abcam, 1:1,000) or α-Tubulin (sc-23948, Santa Crzu, 1:5,000) antibodies. The next day, the membranes were washed with TBS three times and then incubated in the appropriate HRP-conjugated secondary antibody for 30 min. Similarly, the membranes were washed with TBS for three times after secondary antibody incubation. Finally, the immunoreactive bands were detected with an enhanced chemiluminescent substrate (Bio-Rad). The intensities of the bands were quantified with Image-Pro Plus software (v4.5; Media Cybernetics, USA).

### Statistical Analysis

All statistical analyses were conducted on SPSS17.0 software. Data were analyzed by bilateral t test and statistical significance was defined as P < 0.05. The Kolmogorov-Smimov Test and the Levene’s Test were first performed on the two groups of patients. For data with normal distribution and homogeneous variance, independent sample T test was used. Whitney U Test was used for data with non-normal distribution and uneven variance. The chi-square test was used to analyze the correlation of the classified data, and the confounding and influencing factors were analyzed hierarchically. Odds radio (OR) >1 indicates risk factors. Finally, logistic regression model was constructed in variables with P < 0.05.

## Results

### Comparison of Ovarian Response–Associated Risk Factors

A total of 767 women were included in the study, of which 166 showed POR, 409 showed normal ovarian response, and 192 showed HOR. Hierarchical analysis was used for the factors that may cause poor or HOR, and the calculated OR value was listed in detail (**Supplementary Tables 1**, **2**). Since the number of menstrual days and the years of infertility did not conform to the null hypothesis of normal distribution in the poor, hyper, or normal group, the independent sample Mann-Whitney U test and independent sample median test method were used, and the results showed no statistically significant difference (P>0.1) (**Supplementary Tables 1**, **2**). Therefore, it can be considered that the number of menstrual days and years of infertility were not the key factors affecting ovarian reactivity. Our results pointed that age, IVF cycles, BMI, endometriosis, surgical history, and abortion were highly correlated with poor response group compared with these in normal response group (**Table 1**). Multifactor and non-conditional logistic regression analyses were carried out for the factors influencing ovarian response, during which the least significant variables were eliminated gradually, with age ≥35 years old, endometriosis of stage III and IV, abortion ≥4 times finally entering regression equation. Coefficients of the four were positive and significant, indicating there was an association between the four potential risk factors and poor response, as shown in **Table 1**. Similarly, multifactor and non-conditional logistic regression analyses were also carried out for HOR-associated factors, and the results showed that PCOS, underweight, protective factors, and ≥35 years old were correlated with high response (**Table 1**). Age, irregular menstrual cycles, and PCOS were highly correlated with hyper-response group. Intriguingly, age was considered as the risk factor in both poor and hyper-response groups. However, age was positively correlated with poor response group, whereas negatively correlated with hyper-response groups.

**Table 1:** Logistic regression analysis results of statistically significant variables.

Grouping	Variables	B value	S.E.	Odds ratio (OR) (95% confidence interval)	*P* value
	Age ≥ 35 years old	1.313	0.2	3.717 (2.509, 5.505)	0.000
Poor response (n=166)	Endometriosis Stage III	1.562	0.678	4.768 (1.261, 18.021)	0.021
	Endometriosis Stage IV	1.643	0.49	5.173 (1.981, 13.507)	0.001
	Abortion times ≥4	1.218	0.625	3.383 (0.994, 11.503)	0.051
	Age ≥ 35 years old	−0.586	0.247	0.556 (0.343, 0.904)	0.018
Hyper response (n=192)	Underweight	0.695	0.35	2.003 (1.009, 3.977)	0.047
	PCOS	0.773	0.284	2.166 (1.241, 3.781)	0.007

### Relationship Between Different Ovarian Response in IVF-ET and Clinical Outcomes

A retrospective analysis was performed on 200 cases of poor, 200 cases of normal, and 200 cases of high ovarian response patients receiving routine IVF-ET treatment (excluding ICSI treatment cycle) in reproductive medical center of our hospital during 2014–2015 due to tubal factors. The characteristics of patients are shown in **Table 2**. In different response group, the populations were subgrouped according to the age more or less than 35 years old. In the age more than 35 years old groups, we found that the reutilization rate was lower in the hyper-response group, whereas there was no difference in the poor response group compared with these in the normal group (**Table 2**). Meanwhile, the number of valid embryos was higher in the hyper-response group but lower in the poor-response group compared to these in the normal group (**Table 2**). In the group of age less than 35 years old, we demonstrated that there was no difference in hyper- and poor-response groups. However, the number of valid embryos was higher in the hyper-response group but lower in the poor-response group when compared to the normal group (**Table 2**). Clinical outcome analysis results showed that POR with age <35 years old showed significant decline in the numbers of MII oocytes, valid embryos, and clinical pregnancy rate compared to the normal-response group. While numbers of MII oocytes, fertilization rate, valid embryos, and cancelation rate of IVF cycle were higher in the hyper-response group than in the normal group and transferred embryos was less, no significant difference was detected in clinical pregnancy rate between the two (**Table 3**). When it comes to ≥35 years old group, number of MII oocytes, valid embryos, transferred embryos, and clinical pregnancy rate were obviously lower in the poor-response group than in the normal group. The number of MII oocytes and valid embryos in the hyper-response group was significantly increased compared with those in the normal group, and the number of transferred embryos and clinical pregnancy rate were decreased (**Table 3**).

**Table 2:** Laboratory results of different ovarian response groups.

Age	Group	Number of MII oocytes	Fertilization rate	Valid embryos number
< 35 years old	Poor response (n=166)	2.45 ± 1.28*	0.640 ± 0.27	1.74 ± 1.01*
	Normal response (n=409)	6.05 ± 3.29	0.598 ± 0.24	3.396 ± 2.17
	Hyper response (n=192)	10.72 ± 5.40*	0.539 ± 0.23*	5.99 ± 4.35*
≥ 35 years old	Poor response (n=166)	2.17 ± 1.25*	0.626 ± 0.29	1.74 ± 1.02*
	Normal response (n=409)	6.25 ± 2.48	0.630 ± 0.19	3.61 ± 1.82
	Hyper response (n=192)	10.13 ± 5.22*	0.563 ± 0.24	5.50 ± 3.55*

*Means compared with the normal-response group, significant difference between the two groups exists.

**Table 3:** Clinical outcomes of different ovarian response groups.

Age	Group	Number of transplanted embryos	Number of cycle canceled and its rate (%)	Number of biochemical pregnancy and its rate (%)	Number of clinical pregnancy and its rate (%)	Number of abortion and its rate (%)	Number of live birth and its rate (%)
<35 years old	Poor response (n=166)	1.50 ± 0.836	16 (15.39%)	1 (1.14%)	32 (40.32%)^*^	3 (7.69%)	33 (37.5%)
	Normal response (n=409)	1.68 ± 0.858	28 (17.07%)	1 (0.74%)	66 (48.53%)	9 (13.64%)	52 (38.24%)
	Hyper response (n=192)	1.001 ± 0.983^*^	73 (42.94%)^*^	3 (3.09%)	52 (53.61%)	11 (21.15%)	40 (41.24%)
≥35 years old	Poor response (n=166)	1.54 ± 1.03^*^	18 (18.75%)	3 (3.85%)	20 (25.64%)^*^	7 (35%)	10 (29.23%)
	Normal response (n=409)	2.17 ± 1.08	5 (13.89%)	2 (6.45%)	18 (58.065%)	5 (27.78%)	12 (38.71%)
	Hyper response (n=192)	1.67 ± 1.08^*^	4 (13.33%)	1 (3.85%)	7 (26.92%)^*^	1 (14.29%)	5 (19.23%)^*^

*Means compared with the normal-response group, significant difference between the two groups exists.

### The Concentrations of GDF-8 in Different Ovarian Response Patients

**Figure 1:**
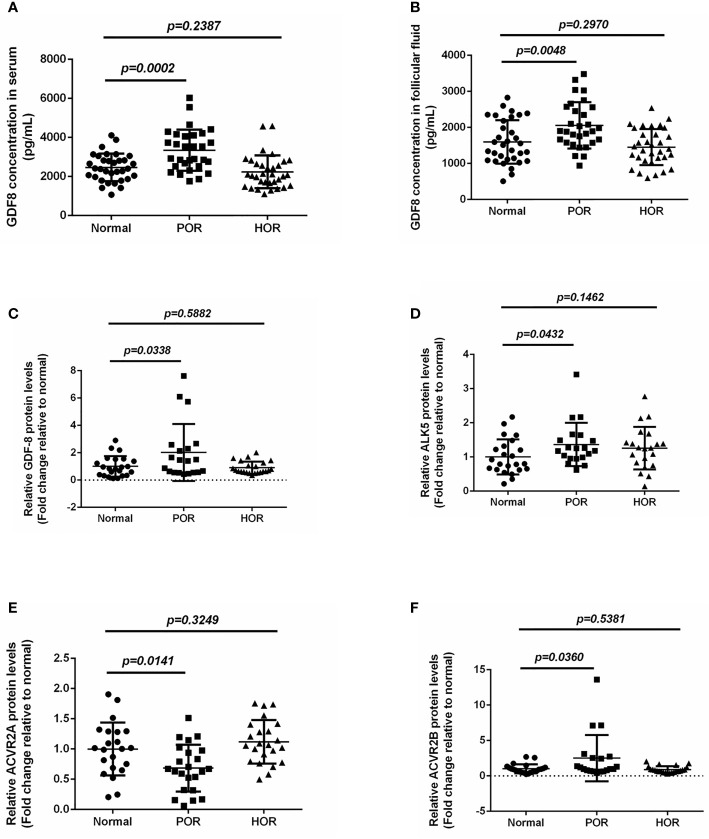
The expression changes of GDF-8 and its responsible receptors in different ovarian response patients. **(A, B)** The accumulation level changes of GDF-8 in serum and follicular fluid obtained from different ovarian response patients were determined by ELISA. **(C–F)** The protein expression level changes of GDF-8 and its responsible receptors (ALK5, ACVR2A, and ACVR2B) were examined in different ovarian response patient granulosa cells using western blot. The data were analyzed by the two-sample t test assuming unequal variances. *P < 0.05* was considered statistically significant. POR (n=30), poor ovarian response. HOR (n=33), hyper ovarian response. Normal, n=33.

Numerous growth factors play essential roles in folliculogenesis *via* autocrine/paracrine manners. The levels of various growth factor are dynamically changing at different follicle development phase. The aberrant expression of growth factors would disrupt the balance of follicular microenvironment and subsequently impair the ovarian response. Accordingly, the changes of follicular growth factor level might be possible indicators of ovarian response during COH process. Exploring the potential ovarian response–related biomarkers will be indispensable for achieving a precise estimation of ovarian response. To evaluate the changes of GDF-8 level in different ovarian response patients, both serum and follicular fluid GDF-8 concentrations were determined by ELISA. Our results showed that both serum and follicular fluid levels of GDF-8 in POR groups were significantly higher than those in the normal-response groups (**Figures 1A, B**). However, there were no significant differences between normal- and hyper-response groups (**Figures 1A, B**).

### The Expression Levels of GDF-8 in Human Granulosa Cells From Different Ovarian Response Patients

The activation of GDF-8-mediated signaling pathway is reliant on the combination of GDF-8 and its putative cellular receptors. It has been reported that GDF-8 activates the downstream signaling pathway by binding to ALK5, ACVR2A, and ACVR2B (7). Our previous study has demonstrated that GDF-8 and its putative cellular receptors ALK5, ACVR2A, and ACVR2B are distributed in intra cells of the follicle (7). To compare the difference of GDF-8 and its cellular receptor expression in human granulosa cells between different ovarian response patients, the protein levels of GDF-8 and its cellular receptors were examined by western blot. Our results found that GDF-8, ALK5, and ACVR2B protein levels in POR patients were higher than those in normal-response patients, whereas there was no difference between the hyper-response and normal-response groups (**Figures 1C, D, F**). Intriguingly, compared with normal-response patients, ACVR2A protein levels in POR patients were lower, and no difference was observed between hyper-response and normal-response patients (**Figure 1E**).

### The GDF-8 Levels Are Negatively Correlated With Ovarian Response

**Figure 2:**
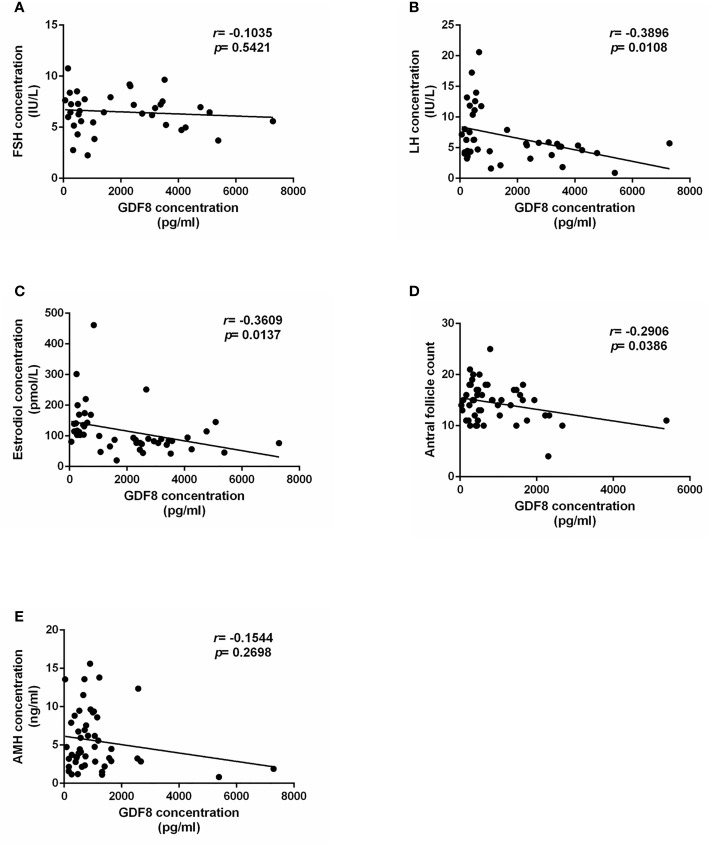
The correlation analysis of GDF-8 and clinical ovarian response patients. **(A–C)** The correlation analysis of GDF-8 levels with hormone concentration, including FSH, LH, and estradiol. **(D)** The correlation analysis of GDF-8 levels with antral follicle count. **(E)** The correlation analysis of GDF-8 levels with AMH levels. The data were analyzed by the two-sample t test assuming unequal variances. *P < 0.05* was considered statistically significant. n=51.

To explore the correlation of GDF-8 with ovarian response, clinical information of patients undergoing IVF were collected. The correlation analysis results showed that GDF-8 levels were negatively correlated with LH and estradiol levels and antral follicle count (**Figures 2B–D**). However, there were no correlation between GDF-8 and FSH, AMH concentration (**Figures 2A, E**).

## Discussion

The female primordial follicle pool, established at birth, contains one million primordial follicles, thereby constituting primary ovarian reserve. However, only approximately 40,000 follicles exist when menarche occurs in adolescent as a result of atresia and degradation of most follicles during childhood. In each menstrual cycle, a number of primordial follicles are recruited and develop into growing follicles, but only one dominant follicle could be mature and then ovulated. Unfortunately, the number of primordial follicles would experience a double decline after the age of 40 and ultimately eliminated at menopause. Thus, the ovarian reserve of women is gradually reducing with enhancement of age. In our present study, we also demonstrated that ovarian response was negatively correlated with women age, in which ovarian response became worse with the increasing age. The negative correlation of both ovarian response and ovarian reserve with aging underpins the view that ovarian reserve is able to partially predict the ovarian response (4). Meanwhile, our results also revealed that young women were more likely to suffer from hyper response. Besides, previous studies have elucidated that overweight is related to decreased ovarian response, implying obesity as a risk factor affecting ovarian response (13). Our data also confirmed that obesity (BMI ≥25 kg/m^2^) was a high risk factor of POR. Interestingly, it has been reported that high BMI does not affect the pregnancy outcomes of IVF in women, derived from the fact that the quality of oocyte obtained by COH has no significant difference between normal and high BMI women (14).

Polycystic ovary syndrome (PCOS) is a common gynecological endocrine disease in women of childbearing age, with an incidence of 5–10% worldwide. Meanwhile, PCOS is a complicated disease affecting multiple systems including the reproductive system. PCOS patients have abnormal follicular development, which is mainly manifested as excessive numbers of follicles in the early growth stage. PCOS patients have more small follicles with stagnant development, and they show a lower FSH threshold, which means a lower level of FSH stimulation is required for these patients to promote follicle regeneration and development (15). Our study demonstrated that PCOS was a high-risk factor for HOR, suggesting that PCOS patients are highly sensitive to gonadotropin. Furthermore, we also demonstrated a negative correlation between irregular menstrual cycles and POR, a condition that otherwise positively correlated with high response. Irregular menstrual cycle is one of PCOS diagnosis bases according to Rotterdam criteria (16). Our results further confirmed the positive correlation between PCOS and HOR. Endometriosis is another gynecological disease that severely endangers women’s reproductive health. It was reported that endometriosis has a negative effect on ovarian reserve and response but shows little influence on pregnancy and live birth rate of IVF (17). Similarly, our present study also demonstrated the potential correlation of endometriosis and ovarian response, in which stages III and IV of endometriosis were positively related to POR, indicating that patients with the abovementioned two stages of endometriosis were more likely to show poor response. Additionally, our results also demonstrated that surgery history, especially pelvic-associated surgery including ovarian and fallopian tube operation, was positively correlated with ovarian response. Surgery for intraovarian diseases, such as ovarian cysts removal, in which electrocoagulation and hemostasis are applied on the ovarian wound, will directly destroy the ovarian tissue, causing damage to the ovarian function and ovarian reactivity. Concomitantly, clinical investigations have underpinned the concept that gynecological surgery is a pivotal factor impairing women ovarian reserve and response, as the consequence of deterioration in ovarian blood supply (18–20), which is intimately associated with the fallopian tube blood condition. Gynecological surgeries, especially salpingectomy, may affect the blood supply of ovary and subsequently lead to the downregulation of ovarian reserve and response. Indeed, our current study demonstrated surgery history was a risk factor of poor response.

It has been debated for decades whether the IVF cycles affect ovarian response. In clinical practice, the exogenous Gn is a widely used stimulatory drug to promote maximum recruitment and maturation of small follicles. Generally, the dose usage of Gn is often far beyond the normal physiological level, which accelerates the depletion of the primordial follicles, thus leading to the decline of ovarian reserve and response. This view is supported by a clinical case that a young infertile patient suffered from continuous decline of ovarian reserve after six consecutive IVF procedures within 4 years (21). Our present study suggested that four or more IVF cycles was a high-risk factor for POR.

The success of IVF-ET depends on the adequate follicle recruitment and maturation, achieved by the means of COH procedure. The proper ovarian response is a major determinant to the outcome of IVF-ET. POR would lead to less follicle recruitment and maturation, whereas HOR patients have an increased risk of developing ovarian hyperstimulation syndrome (OHSS) (5, 22). Our present study found a decrease in clinical pregnancy rates when it comes to patients with POR. Meanwhile, we also found that poor clinical outcomes are subjected to POR *via* imposing negative impact on the number of transplantable embryos and valid embryos. A cohort of investigations on COH patient has reported that HOR has no effect on quality of embryos, embryo implantation, and clinical pregnancy rate when compared to the normal ovarian response group (23, 24). In our study, we demonstrated that HOR population with <35 years old had a higher cancelation rate of IVF cycles, whereas a similar pregnancy rate compared with the control group, which was in accordance with the previous studies (23, 24). Meanwhile, we found a lower pregnancy rate in HOR patients with age ≥35. The diverse pregnancy outcomes in different ages are, at least in part, likely attributed to the changes of endometrial receptivity. The high estrogen levels persistently stimulate the endometrium, leading to the insufficient endometrial secretory phase transformation and subsequently a decrease of pregnancy rate *via* inhibiting endometrial receptivity (25). Additionally, it should be claimed that our present study was based on the clinical information from a single clinical center. The large-scale investigation involved in multicenter clinical evaluation is needed to further confirm our results.

Cell–cell interactions and communications between oocyte and its supporting somatic cells play a crucial role in folliculogenesis. Oocyte and granulosa cell-derived cytokines and growth factors participate in follicle growth and maturation through autocrine/paracrine patterns (6). Multiple growth factors have been reported to regulate folliculogenesis. TGF-β is a well-studied growth factor superfamily encompassing more than 40 members. Within the ovary, TGF-β superfamily members are expressed in divergent cell types. AMH, a granulosa cell-derived growth factor, has been regarded as a reliable biomarker to predict the ovarian reserve and response (26). The studies on TGF-β superfamily member in ovarian physiology reveal the potential value of this functional superfamily in clinical application with an attempt to predict the ovarian response during COH process. Nevertheless, few studies have reported the possibility of other TGF-β superfamily members in evaluating ovarian response. In our current study, we explored the expression changes of GDF-8 in different ovarian response groups. GDF-8 levels were higher in POR groups, indicating GDF-8 was negatively correlated with POR. Importantly, we also demonstrated that the GDF-8 levels are negatively correlated with ovarian response, including LH and estradiol concentration, and antral follicle count. Our results provide a new insight on the possibility of GDF-8 regrading as a potential biomarker of the ovarian response during COH process. More recently, several studies have reported another dimension of the role of GDF-8 in modulating ovarian granulosa cell function (6, 11, 12). In particular, GDF-8 could influence the effects of gonadotropins by regulating their receptors expression (9). Meanwhile, the involvement of GDF-8 in PCOS has also been demonstrated, which underpins the function of GDF-8 in regulating folliculogenesis. In addition, the expression difference of responsible GDF-8 cellular receptors in granulosa cells were also investigated in our present study. Thus far, seven type I and five type II TGF-β superfamily receptors have been identified in mammalian cells. Once binding with the TGF-β superfamily member, the type I receptors will be phosphorylated and subsequently activate the downstream SMAD signaling pathway *via* inducing the phosphorylation. ALK5, ACVR2A, and ACVR2B have been identified as the targets of TGF-β type I and II receptors, mediating the function of GDF-8 in human granulosa cells (27, 28). Our results demonstrated that expression levels of ALK5 and ACVR2B in POR patients were higher compared with those in control groups. However, ACVR2A expression levels in POR patients were lower when compared to the control groups. Taken together, our results demonstrated a negative correlation between GDF-8, in concert with its responsible receptors, and POR. GDF-8 and its receptors appear to be the potential indicators for POR during COH.

In conclusion, our current study demonstrated the potential risk factors involved in different ovarian response during COH. Aging, obesity, endometriosis, surgery history, IVF treatment were the high-risk factors of POR, while irregular menstrual cycles and PCOS were the high-risk factors of HOR. Furthermore, POR patients had a decreased number of MII oocytes and available embryos, resulting in a lower clinical pregnancy rate. The levels of GDF-8, ALK5, and ACVR2B in POR patients were higher compared with those in control groups, whereas ACVR2A expression levels in POR patients were lower. Our study offers a new insight of risk factors correlated with ovarian response and highlights the potential role of GDF-8 level in indicating the ovarian response during COH process.

## Data Availability Statement

The original contributions presented in the study are included in the article/**Supplementary Material**. Further inquiries can be directed to the corresponding authors.

## Ethics Statement

The studies involving human participants were reviewed and approved by the Ethical Committee of the Women’s Hospital, Zhejiang University School of Medicine, China. The patients/participants provided their written informed consent to participate in this study.

## Author Contributions

LB, XY, and YMZ conceived the project and designed the experiments. LB and HHP performed and analyzed the bulk of the experiments. QQC and YJZ performed collection of clinical sample and patient information. YJZ and YX helped perform the experiments. LB and HHP wrote the manuscript, and XY and YMZ revised it. All authors contributed to the article and approved the submitted version.

## Funding

This research was supported by the National Key Research and Development Program of China (No. 2018YFC1003201), the Natural Science Foundation of China (No. 81873819) to YMZ, and the Natural Science Foundation of China (No. 81901443) to LB.

## Conflict of Interest

The authors declare that the research was conducted in the absence of any commercial or financial relationships that could be construed as a potential conflict of interest.

## Publisher’s Note

All claims expressed in this article are solely those of the authors and do not necessarily represent those of their affiliated organizations, or those of the publisher, the editors and the reviewers. Any product that may be evaluated in this article, or claim that may be made by its manufacturer, is not guaranteed or endorsed by the publisher.
