# Supplementary material for: The Exploration of Poor Ovarian Response–Related Risk Factors: A Potential Role of Growth Differentiation Factor 8 in Predicting Ovarian Response in IVF-ET Patient
Source: Front Endocrinol (Lausanne). 2021 Sep 24;12:708089. doi: 10.3389/fendo.2021.708089 (PMC8499678; doi:10.3389/fendo.2021.708089)
Supplement: Supplementary file 1 [file Table_1.docx]

**Supplementary tables**

**Table 1 Analysis of risk factors of poor ovarian response**

| **Grouping** | **Poor**  (n=166) | **Normal**  (n=409) | **Odds ratio (OR)**  **(95% confidence interval)** | ***P* value** |
| --- | --- | --- | --- | --- |
| **Age** |  |  |  |  |
| <35years old | 82（49.4%） | 316(77.3%) | 1 |  |
| ≥35years old | 84（50.6%） | 93(22.7%) | 3.481（2.375, 5.101） | 0.001 |
| **IVF cycles** |  |  |  |  |
| 1 | 97(58.5%) | 252(60.9%) | 1 |  |
| 2 | 51(30.7%) | 123(30.1%) | 1.064（0.712, 1.591） | 0.76 |
| 3 | 10(6.0%) | 32(7.8%) | 0.802（0.380, 1.695） | 0.56 |
| 4 or more | 8(4.8%) | 5(1.2%) | 4.107（1.311, 12.864） | 0.01 |
| **Weight** |  |  |  |  |
| normal | 107(55.73%) | 244(59.3%) | 1 |  |
| underweight | 17(8.85%) | 19(4.6%) | 1.360（0.593, 3.120） | 0.46 |
| overweight | 40(20.83%) | 79(19.3%) | 1.235（0.771, 1.980） | 0.38 |
| obese | 28(14.59%) | 67(16.4) | 1.628（1.019, 2.601） | 0.04 |
| **Endometriosis** |  |  |  |  |
| Not observed | 144（86.8%） | 385（94.1%） | 1 |  |
| Endometriosis Stage I | 5(3.0%) | 10（2.4%） | 1.337（0.449, 3.978） | 0.60 |
| Endometriosis Stage II | 0 | 2（0.5%） | 0.000 | 0.99 |
| Endometriosis Stage III | 6（3.6%） | 4（1%） | 2.659 (1.116, 14.418) | 0.03 |
| Endometriosis Stage IV | 11(6.6%) | 8（2%） | 3.676 (1.450) | 0.01 |
| **PCOS** |  |  |  |  |
| No | 164（98.8%） | 380（92.9%） | 1 |  |
| Yes | 2（1.2） | 29（7.1%） | 0.160（0.038,0.678） | 0.01 |
| **Surgery history** |  |  |  |  |
| No | 95（57.2%） | 275（67.2%） | 1 |  |
| Yes | 71（42.8%） | 134（32.8%） | 1.534（1.059,2.221） | 0.02 |
| **Allergy history** |  |  |  |  |
| No | 150（90.4%） | 371（90.7%） | 1 |  |
| Yes | 16（9.6%） | 38（9.3%） | 1.041（0.564,1.925） | 0.89 |
| **Sexually transmitted diseases** |  |  |  |  |
| No | 165（99.4%） | 409（100%） | 1 |  |
| Yes | 1（0.6%） | 0 | 4.004e9 | 1 |
| **Smoking** |  |  |  |  |
| No | 165（99.4%） | 405（99%） | 0.614(0.068,5.531) | 0.66 |
| Yes | 1(0.6%) | 4（1%） | 0 | 0.99 |
| **Alcohol abuse** |  |  |  |  |
| No | 192（100%） | 409（100%） | 1 |  |
| Yes | 0 | 0 |  |  |
| **Drug abuse** |  |  |  |  |
| No | 192（100%） | 409（100%） | 1 |  |
| Yes | 0 | 0 |  |  |
| **Menarche time** |  |  |  |  |
| 12-15 years old | 148（89.2%） | 358（87.5%） | 1 |  |
| <12 years old | 0 | 0 | - | - |
| >15 years old | 18（10.8%） | 51（12.5%） | 0.854（0.483,1.510） | 0.58 |
| **Menstrual cycles** |  |  |  |  |
| regular | 147(88.6%) | 332(81.2%) | 1 |  |
| irregular | 19(11.4%) | 77(18.8%) | 0.557(0.325,0.955) | 0.03 |
| **Dysmenorrhea** |  |  |  |  |
| No | 117(70.5%) | 267(65.3%) | 1 |  |
| Yes | 49(29.5%) | 142(34.7%) | 0.787(0.533,1.164) | 0.23 |
| **Times of abortion** |  |  |  |  |
| 0 | 84(50.6%) | 198(48.4%) | 1 |  |
| 1 | 40(24.1%) | 123(30.1%) | 0.767(0.494,1.189) | 0.371 |
| 2 | 21(12.7%) | 54(13.2%) | 0.917(0.521,1.613) | 0.538 |
| 3 | 14(8.4%) | 29(7.1%) | 1.138(0.572,2.262) | 3.64 |
| 4 or more | 7(4.2%) | 5(1.2%) | 3.30(1.018,10.69) | 0.47 |

**Table 2 Analysis of risk factors of hyper ovarian response**

| **Grouping** | **Hyper**  (n=192) | **Normal**  (n=409) | **Odds ratio (OR)**  **(95% confidence interval)** | ***P* value** |
| --- | --- | --- | --- | --- |
| **Age** |  |  |  |  |
| <35years old | 167(86.98%) | 316(77.3%) | 1 |  |
| ≥35years old | 25(13.02%) | 93(22.7%) | 0.509(0.315,0.822) | 0.006 |
| **IVF cycles** |  |  |  |  |
| 1 | 114(59.4%) | 252(60.9%) | 1 |  |
| 2 | 64(33.33%) | 123(30.1%) | 1.136(0.781,1.653) | 0.503 |
| 3 | 13(6.77%) | 32(7.8%) | 0.887(0.449,1.754) | 0.731 |
| 4 or more | 1(0.50%) | 5(1.2%) | 0.437(0.050,3.782) | 0.452 |
| **Weight** |  |  |  |  |
| normal | 107(55.73%) | 244(59.3%) | 1 |  |
| underweight | 17(8.85%) | 19(4.6%) | 2.04(1.021,4.079) | 0.044 |
| overweight | 40(20.83%) | 79(19.3%) | 1.155(0.741,1.798) | 0.525 |
| obese | 28(14.59%) | 67(16.4) | 0.953(0.580,1.565) | 0.849 |
| **Endometriosis** |  |  |  |  |
| Not observed | 181(94.27%) | 385(94.1%) | 1 |  |
| Endometriosis Stage I | 4(2.1%) | 10(2.4%) | 0.852(0.263,2.749) | 0.787 |
| Endometriosis Stage II | 2(1%) | 2（0.5%） | 2.127(0.297,15.221) | 0.452 |
| Endometriosis Stage III | 5(2.6%) | 4(1%) | 2.659(0.706,10.019) | 0.149 |
| Endometriosis Stage IV | 0 | 8(2%) | 0 | 0.999 |
| **PCOS** |  |  |  |  |
| No | 164(85.4%) | 380(92.9%) | 1 |  |
| Yes | 28(14.6) | 29(7.1%) | 2.237(1.290,3.880) | 0.004 |
| **Surgery history** |  |  |  |  |
| No | 120(62.5%) | 275(67.2%) | 1 |  |
| Yes | 72（37.5%） | 134(32.8%) | 1.231(0.861,1.761) | 0.254 |
| **Allergy history** |  |  |  |  |
| No | 171(89.1%） | 371(90.7%) | 1 |  |
| Yes | 21(10.9) | 38(9.3%) | 1.199(0.683,2.105) | 0.527 |
| **Sexually transmitted diseases** |  |  |  |  |
| No | 191(99.5%) | 409(100%) | 1 |  |
| Yes | 1(0.5%) | 0 | 3.46E+09 | 1 |
| **Smoking** |  |  |  |  |
| No | 192(100%) | 405(99%) | 1 |  |
| Yes | 0 | 4(1%) | 0 | 0.999 |
| **Alcohol abuse** |  |  |  |  |
| No | 192（100%） | 409（100%） | 1 |  |
| Yes | 0 | 0 |  |  |
| **Drug abuse** |  |  |  |  |
| No | 192（100%） | 409（100%） | 1 |  |
| Yes | 0 | 0 |  |  |
| **Menarche time** |  |  |  |  |
| 12-15 years old | 162（84.4%） | 358（87.5%） | 1 |  |
| <12 years old | 2（1%） | 0 | 3.57E+09 | 0.999 |
| >15 years old | 28（14.6%） | 51（12.5%） | 1.213（0.738,1.994） | 0.446 |
| **Menstrual cycles** |  |  |  |  |
| regular | 138(71.9%) | 332(81.2%) | 1 |  |
| irregular | 54(28.1%) | 77(18.8%) | 1.687(1.130,2.518) | 0.01 |
| **Dysmenorrhea** |  |  |  |  |
| No | 113(58.9%) | 267(65.3%) | 1 |  |
| Yes | 79(41.1%) | 142(34.7%) | 1.315(0.924,1.870) | 0.128 |
| **Times of abortion** |  |  |  |  |
| 0 | 97(50.6% | 198(48.4%) | 1 |  |
| 1 | 50(26%) | 123(30.1%) | 0.83(0.551,1.249) | 0.371 |
| 2 | 31(16.1%) | 54(13.2%) | 1.172(0.708,1.940) | 0.538 |
| 3 | 10(5.2%) | 29(7.1%) | 0.704(0.330,1.503) | 0.364 |
| 4 or more | 4(2.1%) | 5(1.2%) | 1.633(0.429,6.218) | 0.472 |

**Table3 Measurement Data Comparison**

| **Items** | **Null hypothesis** | **Poor respond and normal respond** | | **Hyper respond and normal respond** | |
| --- | --- | --- | --- | --- | --- |
|  |  | **P value** | **conclusion** | **P value** | **conclusion** |
| Menstrual period days | Distribution of menstrual days is the same in two | 0.151 | accept | 0.976 | accept |
|  | Medium time of menstrual days is the same in two | 0.18 | accept | 0.966 | accept |
| Years of infertility | Distribution of infertility time is the same in two | 0.287 | accept | 0.234 | accept |
|  | Medium time of infertility is the same in two | 0.451 | accept | 0.229 | accept |
